# On Diseases of the Liver

**Published:** 1854-08

**Authors:** 


*On Diseases of the Liver.* By GEORGE BUDD, M.D., F.R.S., Professsor of Medicine in King's College, London, and Fellow of Cain's College, Cambridge. Second American, from the last and improved London Edition. With colored plates and Wood Cuts.—Philadelphia, Blanchard and Lea. 1853.

THIS book has been before the medical public several years, and its value is consequently well known. It is one of those practical treatises on the functions and diseases of a particular organ, which embraces the whole subject in detail; and hence it should find a place in the library of every practising physician. It is an octavo volume of 468 pages; and we would especially commend it to the readers of the Journal as a book of practical value. D.

For sale at D. B. Cooke's, Chicago, Ill.

